# Comparison of the effects of litter decomposition process on soil erosion under simulated rainfall

**DOI:** 10.1038/s41598-022-25035-2

**Published:** 2022-12-03

**Authors:** Fangfang Zhu, Jinhua Cheng

**Affiliations:** 1 College of Soil and Water Conservation, Beijing Forestry University, Beijing, 100083 China; 2 Key Laboratory of Soil and Water Conservation and Desertification Control of State Forestry Administration, Beijing, 100083 China

**Keywords:** Hydrology, Forest ecology, Forestry

## Abstract

Overland flow parameters play a pivotal role in soil erosion, which are affected by litter cover in forests. In this study, the litter layer of *Pinus massoniana* (*Masson pine*) was divided into non-decomposed and semi-decomposed layers. Seven litter coverage mass gradients, two slopes (5° and 10°), and two rainfall intensities (60 and 120 mm·h^−1^) were used for a systematic study of the effects of litter layer changes on overland flow dynamic characteristics. The objectives of this study were to explore the soil erosion process in litter different decomposition stages; to explore various relationships between hydraulic variables and litter characteristics. In the process of litter decomposition, overland flow patterns changed from transitional flow to laminar flow and from rapid flow to slow flow. The semi-decomposed layer’s Reynold’s number (*Re*), resistance coefficient (*f*), and soil separation rate (${D}_{r}$) were lower than that of the non-decomposed layer under the same conditions. Litter coverage, runoff and the diameter of the litter were major parameters that affected the *Re*, *f*, *Fr*, and *Dr*. Shrubs with wide leaves should be selected for understory vegetation replanting. The results of this study are helpful to understand the mechanisms of litter influencing erosion processes in different decomposition stages.

## Introduction

Overland flow is a thin layer of flow along slopes under the action of gravity. It is a mixed flow in which turbulent flow and laminar flow coexist, and is typically studied using an open-channel flow research method^1^. Forest canopy layer, grass layer and litter layer are the three active layers of hydrological system. As the second active layer of the forest floor, litter layers is important to the forest hydrological system^2^. The litter layer could be divided into a non-decomposed layer, a semi-decomposed layer, and a completely decomposed layer. Given that litter layers have the functions of water conservation, rainfall retention, and soil moisture maintenance^3–5^, as well as redistribution of rainfall and overland flow, litter has an important impact on forest soil erosion. Scholars have found that litter affects hydrological processes mainly via its composition, characteristics, degree of decomposition, and coverage^6–8^.

The litter layers’ soil and water conservation functions are reflected by two characteristics: storage and interception. That is due to the loose structure of the litter layer, which can reduce the kinetic energy of raindrops and absorb precipitation. The water-holding properties of litter are good^9,10^. Liu et al.^11^ studied the water-holding capacity of litter in needle-leaf forest, broad-leaf forest and mixed forest found that the total maximum water-holding capacity of litters of the three forests ranged from 17.85 to 19.87 t·hm^−2^, and the maximum water-holding rate of litter ranged from 200.6 to 228.0%. The water-holding capacity of litter is mainly played by the non-decomposed and semi-decomposed layers. The maximum water-holding capacity of litter is 1.4–1.7 times larger than its weight, and litter can also intercept rainfall by approximately 2 mm during a rainfall event^12,13^. Zhao et al.^14^ evaluated the impact of two different litter shapes on interception, and the results showed that the average amount of rainfall intercepted by coniferous litter was 2.08 times that of broadleaf litter. Litter interception storage capacity increases with rain intensity in common conditions (under 50 mm·h^−1^), and broad-leaf litter intercepts more rainwater than needle-leaf litter^15^.

In addition, litter layer has good permeability, which can slow down the effect of rainfall on surface soil particles by reducing the kinetic energy of raindrops, slowing the generation and movement of surface runoff, promoting soil infiltration^16^, and reducing overall soil erosion^17–19^. In recent years, experts have paid more attention to the study of the relationship between litter and overland runoff. Litter layers have crucial effect on overland flow. The thickness and coverage of litter are directly proportional to runoff generation time and inversely proportional to flow rate, sediment yield, and overland flow velocity^20,21^. As litter coverage mass increases, the amount of soil loss and runoff decreases^22^. Xia et al.^23^ studied the hydrological effects of the litter layer on the Loess Plateau and found that the litter layer of coniferous and broad-leaved tree species can reduce runoff rates by 19% and 26%, respectively. Meanwhile, Ding and Fu^24^ studied the influence of leaf morphology in the litter layer on sediment transport capacity and determined that long, narrow, and small round leaves can significantly reduce runoff carrying capacity. Therefore, the influence of litter layer on soil erosion is related to its own leaf morphology.

At present, the methods of field observation and indoor rainfall simulation are often used in the research of overland flow, including its runoff velocity, flow pattern discrimination, and resistance coefficient^25^. Rainfall on an intact soil in the field makes it difficult to analyse the effects of individual variables. Therefore, the research on overland flow mostly utilizes indoor simulated rainfall tests^26^. Li et al.^27^ studied the effects of two types of litter on runoff under simulated rainfall and found that the litter reduced runoff and delayed the beginning of runoff. By studying the effects of different vegetation litters on the characteristics of overland flow, Sun et al.^28^ found that litter coverage could significantly reduce the Reynolds number and flow power by 8–29% and 56–80%, respectively. Wang et al.^29^ studied the effects of four types of litter coverage and incorporation on soil erosion and found that the amount of erosion under litter-incorporated treatment was 5.4 times as large as that of litter-covered treatment. As mentioned above, litter affect hydrological and erosion processes greatly. The researchers regard the litter as an ensemble, without distinguishing the litter into different stages. These studies did not systematically study the effects of litter layer on overland flow.

In this study, under simulated rainfall conditions, the influence of the characteristics of a *Pinus massoniana* litter decomposition stage on hydrodynamic characteristic parameters such as flow patterns, flow resistance coefficient, soil detachment capacity was elucidated. The specific aims of this study were to: (a) study the soil erosion process in litter different decomposition stages; and (b) analyse how the differences in soil loss between litter cover vary with litter decomposition stage. This study may enhance the understanding of the mechanisms of litter influencing erosion processes in different decomposition stages.

## Materials and methods

### Study area description

**Figure 1:**
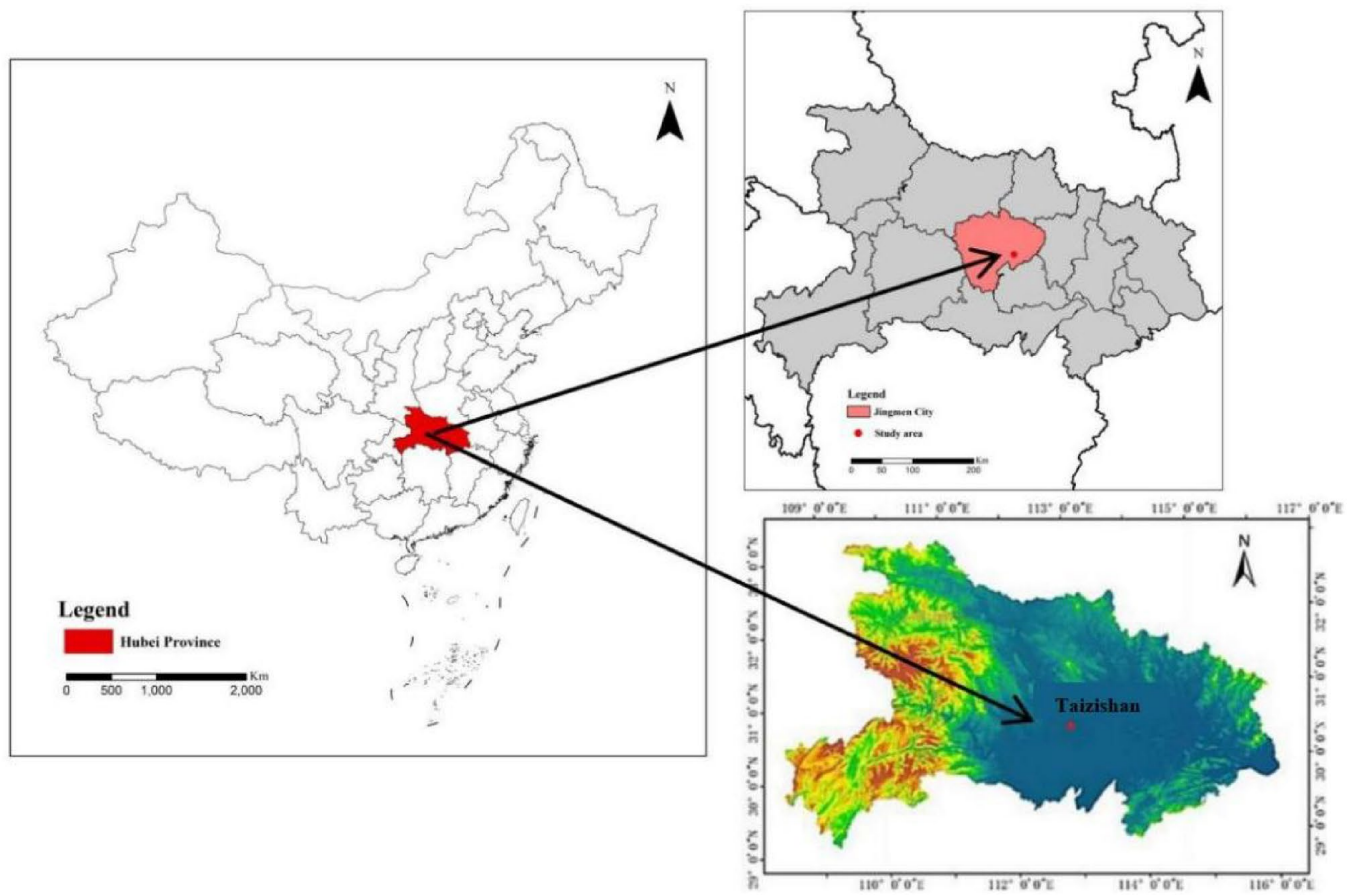
Geographic location of the study area. Maps were generated using ArcGIS 10.8 for Desktop (http://www.esri.com/software/arcgis/arcgis-for-desktop).

Yangtze River Basin is situated in central China (Fig. 1). Its geographical coordinates are between 30° 48′ 30″–31° 02′ 30″ N and 112° 48′ 45″–113° 03′ 45″ E. Taizishan is located in the transition zone between the north and south of China, with an altitude of 403–467.4 m. It belongs to the subtropical monsoon humid climate zone and has obvious karst landforms. The farm area is 7576 hectares, the forest coverage rate is 82.0%, and the vegetation is mainly Masson pine, fir, and various broad-leaved tree species. Increased forest coverage reduces sediment production^30^. The soil is mainly viscous yellow–brown soil and loess parent material. Rain is concentrated in summer, with an average annual rainfall of 1094.6 mm and an average annual temperature of 16.4 °C. Rainfall-related flood risk increased in the Yangtze River Delta in recent years^31^.The study was based in a *Pinus massoniana* forest in the Taizishan forest farm of Hubei Province. The *Pinus massoniana* (Masson pine) is a common species distributed in Central China.

### Experiment design

**Figure 2:**
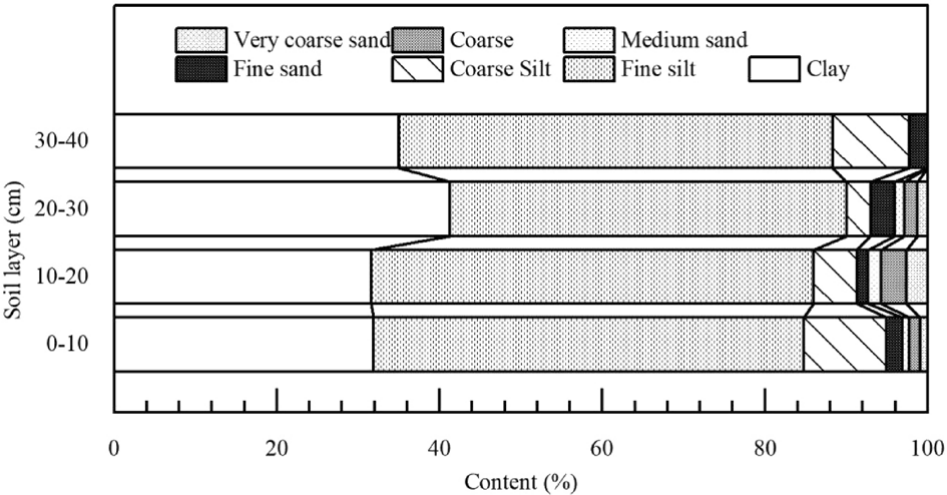
Soil particle composition of study area soil layers.

We chose the *Pinus massoniana* forest with 47a in the study area as the research object. In the typical *Pinus massoniana* forest, the separate layers of litter (semi-decomposed and non-decomposed layers) were collected from several 1 m × 1 m quadrat and placed in grid bags. The litter of the semi-decomposed layer have no complete outline, and the color was brown. As the litter leaves of the completely decomposed layer are powdery and are combined with the soil layer, this layer is difficult to collect. Before testing, it was necessary to clean the soil off the pine needles and then allow the litter to dry naturally. The characteristics of the semi-decomposed and non-decomposed litter layers are shown in Table 1. The soil samples need to be dried and screened by 10 mm. When filling the soil trough, every 0.1 m of soil thickness was one layer, for a total of four layers (0.4 m). The characteristics by soil particle sizes are different (Fig. 2). The soil samples were dried naturally, crushed, and then sieved. The soil trough (2 m long, 0.5 m wide and 0.5 m deep) was filled to have a bulk density of 1.53 g·m^−3^. In this process, an appropriate amount of water was sprinkled on the surface of each soil layer to achieve a soil moisture content consistent with the surrounding, undisturbed, or natural, state. The simulation experiment was conducted in the Jiufeng rainfall laboratory at Beijing Forestry University, China. We used a rainfall simulation system (QYJY-503T, Qingyuan Measurement Technology, Xi’an, China) used a rotary downward spray nozzle. The system is able to simulate a wide range of rainfall intensities (10 to 300 mm h^−1^) using various water pressure and nozzle sizes controlled by a computer system.

**Table 1:** Characteristics of the non-decomposed and semi-decomposed layers of *Pinus massoniana* litter.

Litter characteristics	Non-decomposed layer	Semi-decomposed layer
Length/(cm·g^−1^)	27.26	16.24
projected area/(cm^2^·g^−1^)	7.06	6.28
Surface area/(cm^2^·g^−1^)	147.62	95.41
Average diameter/mm	0.84	0.28
Litter density/(g·cm^−3^)	0.53	0.76
Maximum water-holding capacity/(t·hm^−2^)	4.46	6.97
Stock volume/(t·hm^−2^)	4.28	3.17

Litter characteristics were determined by Win RHIZO Pro 2.0 image analysis software, and litter density measured by a volumetric drainage method.

According to the results of the field forest investigation, the litter was covered with the experimental treatments shown in Table 2. The treatments mass coverage of non-decomposed litter layer was named as follows: N1 denoted litter mass coverage 0 g·m^−2^, N2 was ‘the non-decomposed litter mass coverage 100 g·m^−2^’, N3 was ‘the non-decomposed litter mass coverage 200 g·m^−2^’, and N4 was ‘the non-decomposed litter mass coverage 400 g·m^−2^’, N5 was ‘the semi-decomposed litter mass coverage 100 g·m^−2^’, N6 was ‘the non-decomposed litter mass coverage 100 g·m^−2^ and the semi-decomposed litter mass coverage 100 g·m^−2^’, N7 was ‘the non-decomposed litter mass coverage 200 g·m^−2^ and the semi-decomposed litter mass coverage 100 g·m^−2^’. N2, N3 and N4 were the undissolved state of litter layer, and N4 (non-decomposed state, ND), N7 (initial stage of litter decomposition, ID), N6 (middle stage of litter decomposition, MD) and N5 (final stage of litter decomposition, FD) respectively represent different stages of litter decomposition.

**Table 2:** The experimental design of this study.

Litter mass coverage/(g·m^−2^)	Treatment number						
	N1	N2	N3	N4 (ND)	N5 (FD)	N6 (MD)	N7 (ID)
Semi-decomposed layer	0	0	0	0	100	100	100
Non-decomposed layer	0	100	200	400	0	100	200
Total	0	100	200	400	100	200	300

In this study, biomasses of the non-decomposed and semi-decomposed layers of litter were combined, and then divided into seven treatments (N1–N7).

*ND* is non-decomposed state, *ID* is initial stage of litter decomposition, *MD* is middle stage of litter decomposition, *FD* is final stage of litter decomposition.

According to the rainfall in the Taizishan area of Hubei Province, erosive rainfall and extreme rainstorms were selected as the research conditions. Summer rainfall events occur mainly in the summer in this area, and a rainfall intensity of 60 mm·h^−1^ was the most common erosive rainfall intensity. Under extreme weather conditions, the rainfall intensity can reach up to 120 mm·h^−1^. Our experiments were conducted with 60 and 120 mm·h^−1^ rain intensities with a rainfall that lasted 1 h. According to the field investigation data of forest land, this area is a low mountain and hilly area with a slope mostly between 5° and 10°. Therefore, 5° and 10° were selected for the slope treatments in this study. The combination of slope and rainfall intensity was named as follows: T1 denoted ‘Slope 5° and rainfall intensity 60 mm·h^−1^’, T2 was ‘Slope 10° and rainfall intensity 60 mm·h^−1^’, T3 was ‘Slope 5° and rainfall intensity 120 mm·h^−1^’, and T4 was ‘Slope 10° and rainfall intensity 120 mm·h^−1^’. With two rainfall intensities, two slopes, seven litter coverage gradient and two repetitions combined, this study had a total of 56 rainfall events.

### Experimental procedure

Before the test, the soil samples were wetted for 10 h and then drained for 2 h to eliminate the effect of the initial soil moisture on the soil detachment measurement. When the simulated rainfall started, all the runoff and sediment produced from plot were collected every 5 min in the first 10 min, and then collected once every 10 min during the subsequent 50 min. At the same time, runoff velocity, depth and temperature were measured and vernier calliper (accuracy 0.02 mm) respectively.

The overland flow velocity was measured using dying method (KMnO_4_ solution)^32^. After judging the flow pattern, we confirmed the correction coefficient *K* value (in laminar flow state, *K* = 0.67; transition flow state, *K* = 0.70; turbulent flow state, *K* = 0.8). The average velocity of overland flow was obtained by multiplying the correction coefficient *K* and the instantaneous velocity. Runoff depth was measured using vernier calliper (accuracy 0.02 mm). Runoff temperature was measured using thermometer. When the rainfall experiment finished, the collected runoff samples were measured volumetric cylinder and then settled for at least 12 h. The clear water was decanted, and the samples were put into an oven to dry for 24 h under 105 °C. The sediment sample was dried and weighed with an electronic scale.

### Calculation of hydrodynamic parameters

Overland flow has the characteristics of a thin water layer, large fluctuations of the underlying surface, and unstable flow velocity. At present, most scholars use open-channel flow theory to study overland flow^33,34^. In open-channel flow theory, the Reynold’s number (*Re*), Froude constant (*Fr*), flow index (*m*), resistance coefficient (*f*), and soil separation rate (${D}_{r}$) are the basic parameters of overland flow dynamics, through Reynold’s number (*Re*), Froude constant (*Fr*), flow index (*m*) can distinguish flow patterns. *Re* is calculated as:

$$Re=R\cdot V/\nu ,$$

where *Re* is the Reynolds number of the water flow, which is dimensionless, and can be used to judge the flow state of overland flow. When *Re* ≤ 500, the flow pattern is laminar; when 500  <  *Re* ≤ 5000, the flow pattern is transitional; when *Re* > 5000, the flow pattern is turbulent. *R* is the hydraulic radius (m), which is generally replaced by flow depth as measured by a vernier calliper (accuracy 0.02 mm). $V$ is the average velocity (m·s^−1^); $\nu$ is the kinematic viscosity coefficient (m^2^·s^−1^), and the calculation formula is $\nu$ = 0.01775·10^−4^·(1 + 0.0337 t + 0.00021 t^2^), where t is the test overland flow temperature^35^.

*Fr* is the Froude constant, which is the ratio of the inertial force to gravity and can be used to distinguish overland flow as rapid flow, slow flow, or critical flow. When *Fr* < 1, the fluid is considered as slow flow; when *Fr* = 1, the fluid is critical flow; when *Fr* > 1, the fluid is rapid flow.

*Fr* is calculated as:

$$Fr=V/\sqrt{g\cdot R},$$

where $Fr$ is the Froude constant of the water flow, which is dimensionless; $V$ is the average velocity (m·s^−1^); *g* is the acceleration of gravity and has a constant value of 9.8 m·s^−2^; *R* is a hydraulic radius (m), and is generally replaced by flow depth as measured by a vernier calliper (accuracy 0.02 mm).

Regression fitting is made for runoff depth (*h*) and single width flow (Q). The runoff depth equation for slope is as follows:

$$h=k{q}^{m},$$

where *q* is the single width flow (L·m^−1^·s^−1^); *h* is the depth of water on the slope (m); and *m* is the flow index, which reflects the turbulent characteristics of the flow state. The larger *m* is, the more energy the flow consumes in the work of resistance. The comprehensive index (*k*) reflects the characteristics of the underlying surface and the water viscosity of the slope flow. The larger *k* is, the stronger the surface material of the slope works on the flow.

The resistance of overland flow reflects the inhibition effect of different underlying surface conditions on the velocity of overland flow. The Darcy–Weisbach formula is widely used in research because of its two advantages: applicability and dimensionlessness under laminar and turbulent flow conditions^36,37^.

The resistance coefficient (*f*) is calculated as follows:

$$f=8\cdot g\cdot R\cdot J/{V}^{2},$$

where the resistance coefficient *f* has no dimension; *g* is the acceleration of gravity and is always 9.8 m·s^−2^; *R* is a hydraulic radius (m), generally replaced by flow depth measured by a vernier calliper (accuracy 0.02 mm); $V$ is the average velocity (m·s^−1^); and *J* is the hydraulic gradient, which can be converted by the gradient in a uniform flow state and is generally replaced by the sine value of the gradient.

Shear stress ($\tau$) is the main driving force that affects the stripping of soil particles from the surface soil^38^. Shear stress is calculated as:

$$\tau =r\cdot g\cdot R\cdot J,$$

where $\tau$ is the shear force of runoff (Pa); and *r* is the density of water and sediment concentration flow (kg·m^−3^). This study used a muddy water mass and volume ratio in the unseparated state to calculate the density of water and sediment concentration flow.

Flow power (*W*) is the runoff power per unit area of water and refers to the power consumed by the weight of water acting on the riverbed surface to transport runoff and sediment. *W* is calculated as:

$$W=\tau \cdot V,$$

where *W* is the flow power (N·m^−1^·s^−1^); and $\tau$ is the shear force of runoff (Pa).

Soil separation rate (${D}_{r}$) refers to the quality of soil in which soil particles are separated from the soil per unit time. The calculation formula is as follows:

$${D}_{r}={W}_{d}-{W}_{w}/t\cdot A,$$

where ${D}_{r}$ is the rate of soil separation (kg·m^−2^·s^−1^); ${W}_{w}$ is the dry weight of soil before the test; ${W}_{d}$ is the dry weight of soil after the test, measured by the drying method (kg); t is the scouring time (s); and *A* is the surface area of the soil sample (m^2^).

## Results

### Variations in overland flow pattern under litter coverage

**Figure 3:**
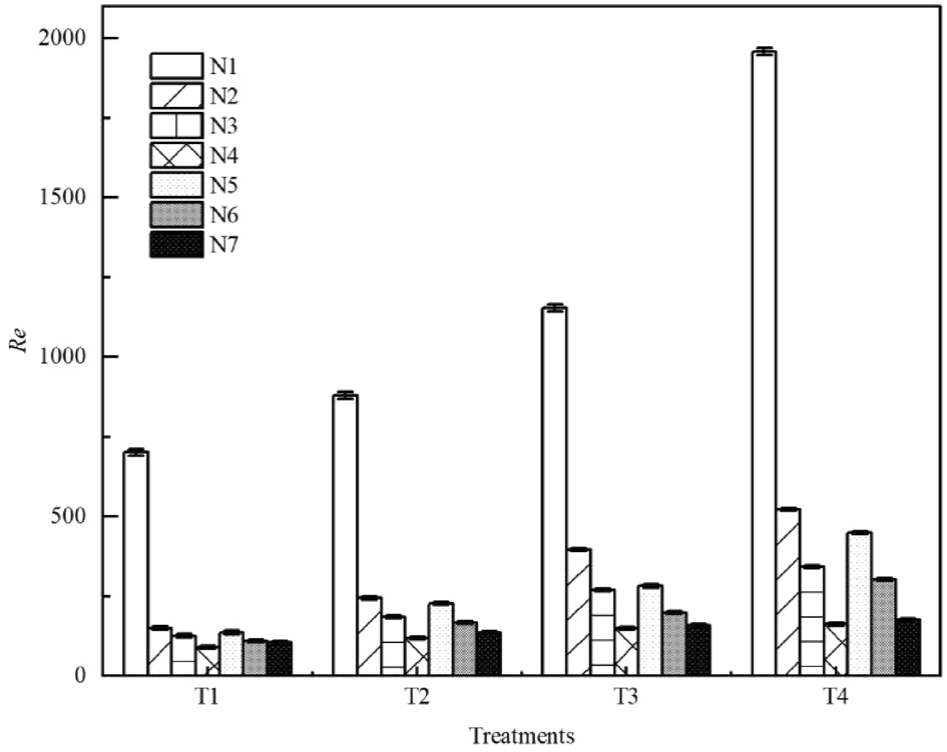
Comparison of the Reynolds number *Re* between seven types of litter layer. *Re* is the Reynolds number of the water flow; N1–N7 are treatments, see Table 2 for treatment specifics; T1 is slope 5°, rain intensity 60 mm·h^−1^; T2 is slope 10°, rain intensity 60 mm·h^−1^; T3 is slope 5°, rain intensity 120 mm·h^−1^; T4 is slope 10°, rain intensity 120 mm·h^−1^. Error bars indicate standard deviation.

In this study, Comparison of the Reynolds number *Re* between seven types of litter layer are shown in Fig. 3. Compared with bare land as the control (N1), the *Re* of overland flow under litter cover decreased by 81%; compared with N2 and N5, the *Re* of overland flow decreased by 72% in the non-decomposed layer and 77% in the semi-decomposed layer. When the rainfall intensity was 120 mm·h^−1^ and the slope was 10° (i.e., T4) the coverage mass of the non-decomposed layer of litter was 0 g·m^−2^ and 100 g·m^−2^, the *Re* is greater than 500 and the overland flow is not laminar flow. Overall, *Re* decreased with the increase in litter coverage mass. *Re* increased gradually in the process of litter decomposition, which reached the maximum at N5 (FD stage). The difference of *Re* between N5 and N6 was the largest, and between N4 and N7 was the smallest. In this study, the *Re* was less than 500 in the decomposition stage (N5, N6, N7), the flow pattern were laminar.

**Figure 4:**
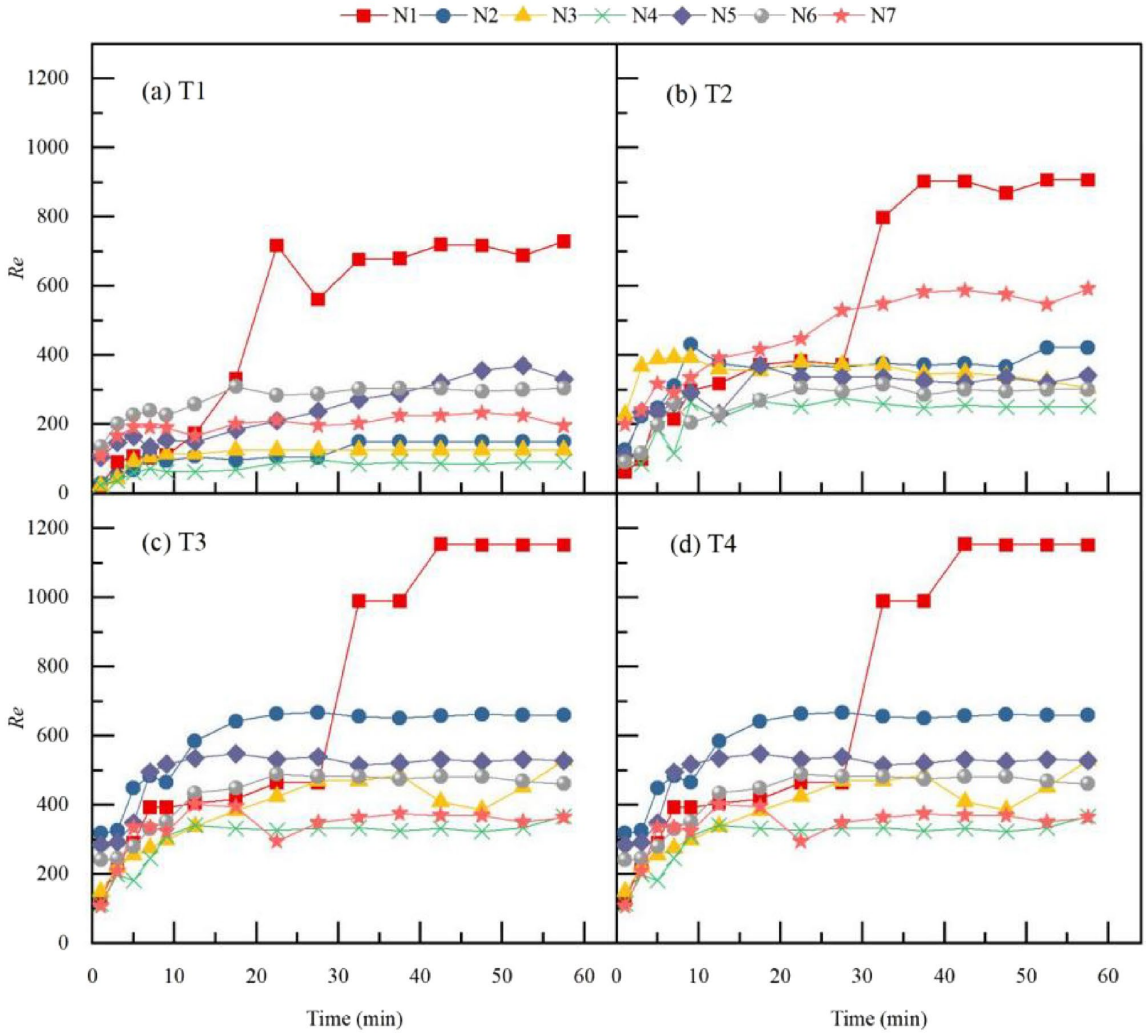
Time series of Reynolds number *Re* for seven types of litter layer. *Re* is the Reynolds number of the water flow; N1–N7 are treatments, see Table 2 for treatment specifics. T1 is slope 5°, rain intensity 60 mm·h^−1^; T2 is slope 10°, rain intensity 60 mm·h^−1^; T3 is slope 5°, rain intensity 120 mm·h^−1^; T4 is slope 10°, rain intensity 120 mm·h^−1^.

The *Re* increased with the increase in rainfall intensity and slope. The larger the litter coverage mass, the less significant the influence of rainfall intensity and slope on *Re*. A comparison of the changes in *Re* for the slopes with or without litter coverage under a state of 10° and 120 mm·h^−1^ (i.e., T4) reveals that, the influence of litter coverage on *Re* is the largest in this treatment condition, and the difference in the *Re* is 219. Time series of Reynolds number *Re* for seven types of litter layer are shown in Fig. 4. With the extension of rainfall duration, *Re* showed a trend of initial rapid growth and then stabilisation.

**Figure 5:**
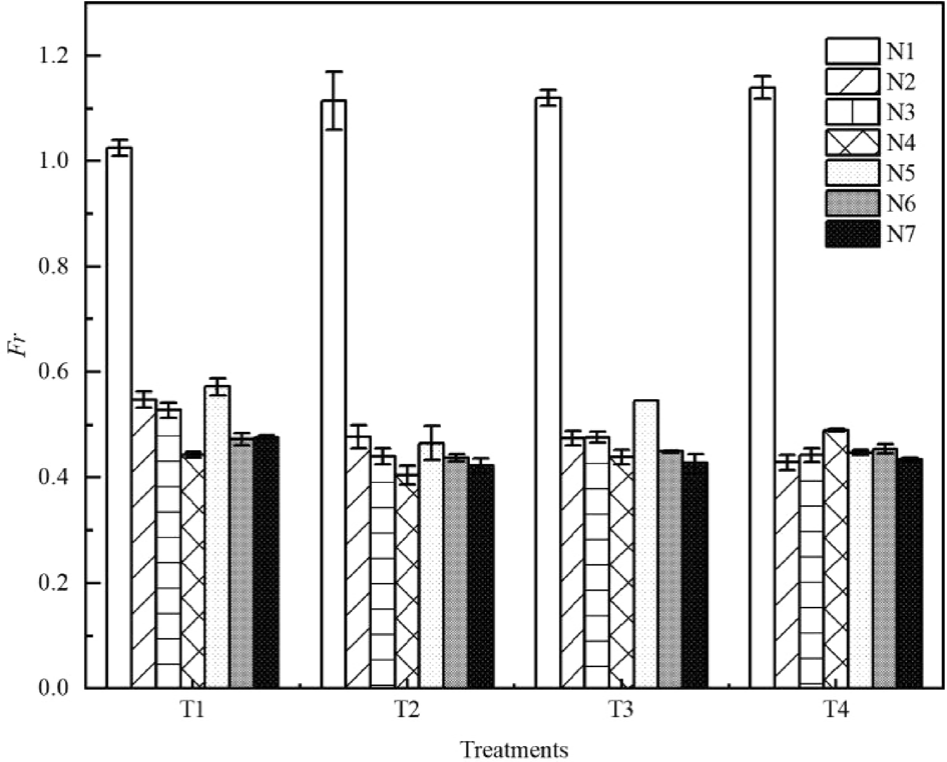
Comparison of the Froude constant *Fr* between seven types of litter layer. *Fr* is the Froude constant; N1–N7 are treatments, see Table 2 for treatment specifics; T1 is slope 5°, rain intensity 60 mm·h^−1^; T2 is slope 10°, rain intensity 60 mm·h^−1^; T3 is slope 5°, rain intensity 120 mm·h^−1^; T4 is slope 10°, rain intensity 120 mm·h^−1^. Error bars indicate standard deviation.

Comparison of the Froude constant *Fr* between seven types of litter layer are shown in Fig. 5. The *Fr* was greater than 1 with the condition of bare land, and thus the overland flow pattern was a rapid flow. As the *Fr* range of overland flow was 0.405–0.548 (all less than 1), the flow pattern with litter coverage was slow flow. Overall, the *Fr* of overland flow decreases with the increase in litter coverage mass. Compared with bare land, the *Fr* of overland flow with litter cover decreased by 63%. A comparison of the effects of the semi-decomposed and non-decomposed layers of litter on the *Fr* with the same litter coverage mass reveals that the *Fr* of overland flow within the semi-decomposed layer is larger than that of the non-decomposed layer. The *Fr* of overland flow under the N6 treatment was smaller than that under the N3 treatment. *Fr* increased gradually in the process of litter decomposition, which reached the maximum at N5 (FD stage).

The relationship between runoff depth (*h*) and unit width flow (*q*) under different litter coverage masses is shown in Table 3, where *m* is the runoff flow pattern index, and *k* is the comprehensive index of the quality characteristics of litter coverage. Comparing the data from the N2 and N5 groups, the values of *k* and *m* under the semi-decomposition litter layer are higher. This result shows that the semi-decomposed litter layer had a more obvious effect on the increase of surface roughness and the change of flow patterns. *m* and *k* increased in the process of litter decomposition, which showed that surface roughness increased in litter decomposition degree. There is a significant correlation between *m* and *k* (p < 0.01), which shows that the overland flow’s bed characteristics are the sole factor that affects the flow turbulence’s characteristics.

**Table 3:** Regression relationships between runoff depth (*h*) and unit width discharge (*q*) with different litter coverage masses.

Treatment	F(x)	R^2^	*k*	*m*
N1	*h* = 0.091 *q*^0.755^	0.935	0.091	0.755
N2	*h* = 0.313 *q*^1.045^	0.857	0.313	1.045
N3	*h* = 0.186 *q*^0.933^	0.776	0.186	0.933
N4	*h* = 0.012 *q*^0.417^	0.776	0.012	0.417
N5	*h* = 0.325 *q*^1.049^	0.772	0.325	1.049
N6	*h* = 0.163 *q*^0.917^	0.831	0.163	0.917
N7	*h* = 0.054 *q*^0.691^	0.944	0.054	0.691

*h* is runoff depth, m, *q* is unit width flow, L·m^−1^·s^−1^, *m* is flow pattern index, *k* is comprehensive index.

### Variations in overland flow resistance coefficient under litter coverage

**Figure 6:**
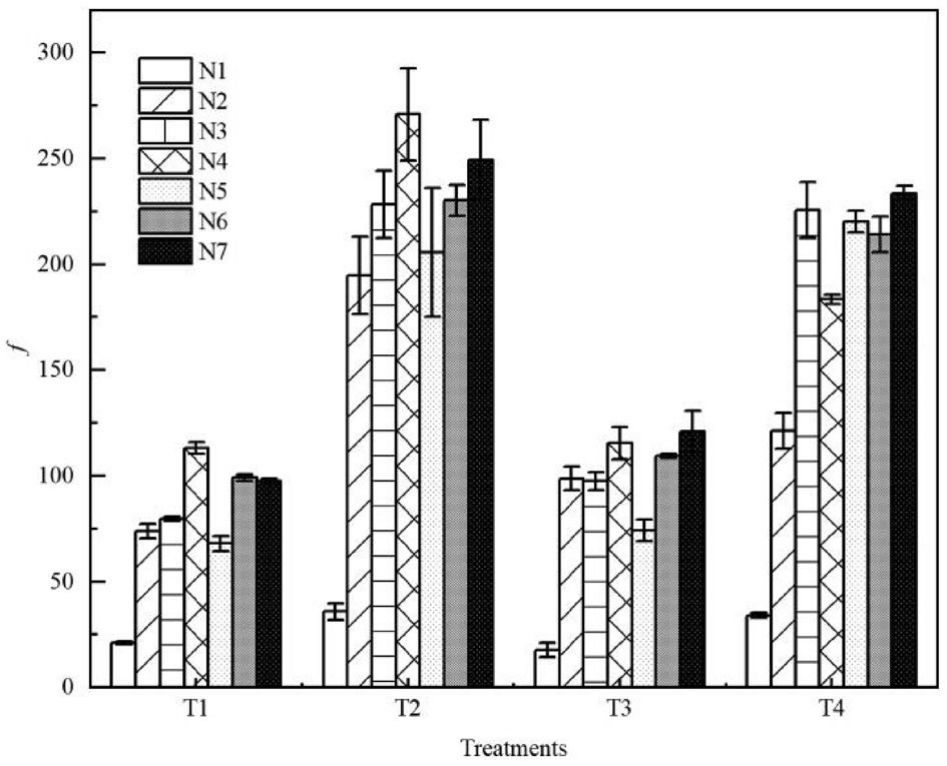
Comparison of the resistance coefficient *f* between seven types of litter layer. *f* is the resistance coefficient; N1–N7 are treatments, see Table 2 for treatment specifics; T1 is slope 5°, rain intensity 60 mm·h^−1^; T2 is slope 10°, rain intensity 60 mm·h^−1^; T3 is slope 5°, rain intensity 120 mm·h^−1^; T4 is slope 10°, rain intensity 120 mm·h^−1^. Error bars indicate standard deviation.

The resistance coefficient (*f*) refers to the resistance in the process of runoff movement. The energy consumed by the overland flow to overcome resistance is directly proportional to the *f* of overland flow under litter coverage, as shown in Fig. 6. The average *f* of *Pinus massoniana* litter coverage was 4.568 times that of bare land. Compared with bare land (N1), the *f* of overland flow was 3.521 and 3.901 times higher for the non-decomposed and semi-decomposed layers, respectively. Compared with the N2 treatment, which had the smallest litter coverage mass, the overland flow *f* of the other treatments with rainfall intensities of 60 and 120 mm·h^−1^ increased 1.23 and 1.41 times on average, respectively. When the rainfall intensity was 60 mm·h^−1^, the *f* increased with the increase in litter coverage mass; the *f* increased with the decomposition process. Nevertheless, with the same litter coverage mass, the *f* of the non-decomposed litter layer was 1.078-fold higher than that of the semi-decomposed layer. When the rainfall intensity was 120 mm·h^−1^, the *f* first increased and then decreased with the increase in litter coverage mass and the deepening of litter decomposition. The *f* of the N6 group was 1.08 times higher than that of N3 under the same litter coverage mass. The semi-decomposed layer of litter could significantly increase the *f* of overland flow (p < 0.01).

### Variations in soil detachment capacity under litter coverage

**Figure 7:**
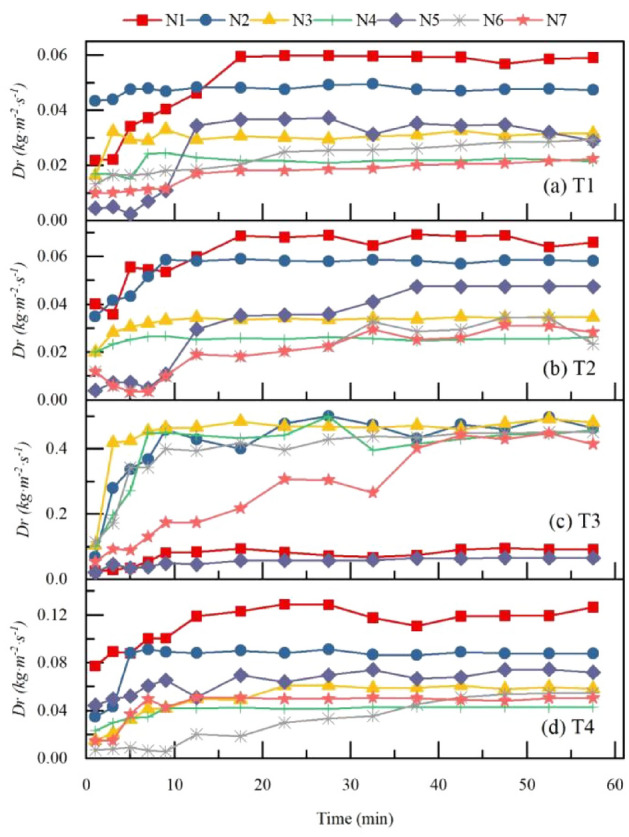
Time series of soil separation rate ${D}_{r}$ for seven types of litter layer. ${D}_{r}$ is the rate of soil separation; N1–N7 are treatments, see Table 2 for treatment specifics; T1 is slope 5°, rain intensity 60 mm·h^−1^; T2 is slope 10°, rain intensity 60 mm·h^−1^; T3 is slope 5°, rain intensity 120 mm·h^−1^; T4 is slope 10°, rain intensity 120 mm·h^−1^.

The main source of sediment in the initial stage of erosion is due to soil separation^39^. Time series of soil separation rate *Dr* for seven types of litter layer. are shown in Fig. 7. In general, *Dr* first increased and then stabilize with rainfall duration. With an increase in rainfall duration, *Dr* increased the most in the N1 treatment compared with all other treatments. From the perspective of overall change, the *Dr* decreases with the increase in litter coverage mass. Compared with bare land, the *Dr* with litter coverage decreased by 47%. Compared with the N2 and N5 treatments, the *Dr* decreased by 22% in the non-decomposed layer and 36% in the semi-decomposed layer. When the rainfall intensity was 60 mm·h^−1^, the soil separation rate increased with the increase in slope. Comparing the soil separation rates of N2 and N5, the *Dr* within the same litter coverage masses were compared: the non-decomposed layer of litter had a larger *Dr* than that of the semi-decomposition layer.

**Figure 8:**
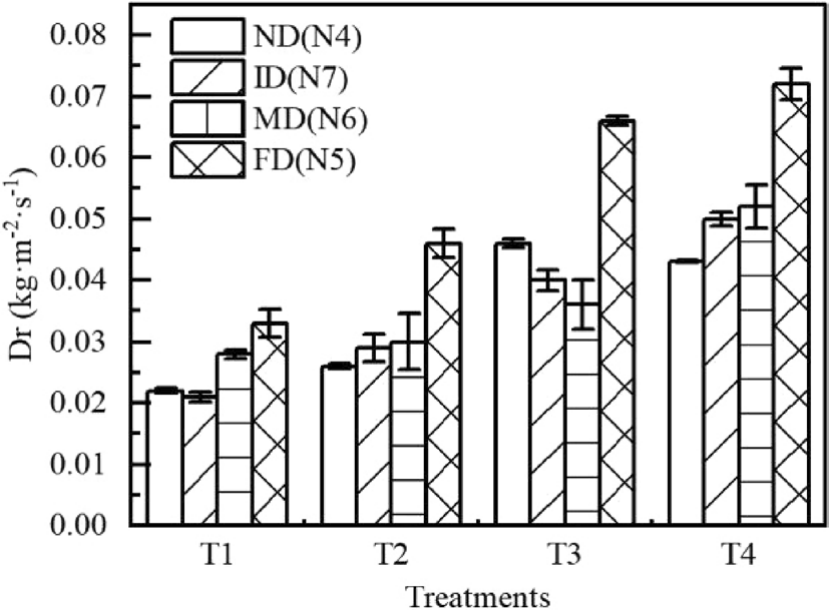
Comparison of soil separation rate ${D}_{r}$ between four litter decomposition stages. ${D}_{r}$ is the rate of soil separation; N1–N7 are treatments, see Table 2 for treatment specifics; T1 is slope 5°, rain intensity 60 mm·h^−1^; T2 is slope 10°, rain intensity 60 mm·h^−1^; T3 is slope 5°, rain intensity 120 mm·h^−1^; T4 is slope 10°, rain intensity 120 mm·h^−1^. *ND* is non-decomposed state, *ID* is initial stage of litter decomposition, *MD* is middle stage of litter decomposition, *FD* is final stage of litter decomposition.

**Figure 9:**
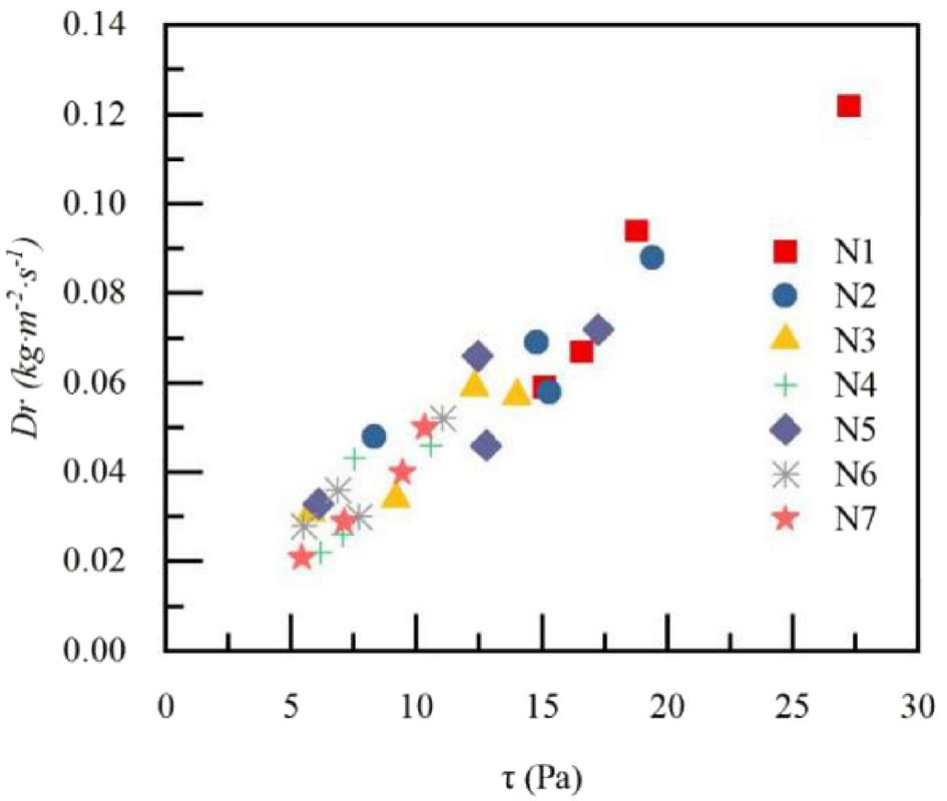
Relationship between shear force of runoff *τ* and soil separation rate ${D}_{r}$ *of* seven types of litter layer. *Dr* is the rate of soil separation; N1–N7 are treatments, see Table 2 for treatment specifics; $\tau$ is the shear force of runoff.

Comparison of soil separation rate between between four litter decomposition stages are shown in Fig. 8. The maximum *Dr* appeared at the final stage of litter decomposition (FD) stage. Except for T3 treatment, the *Dr* gradually increased with the litter decomposition process. Relationship between shear force of runoff τ and soil separation rate *Dr* of seven types of litter layer. is shown in Fig. 9. The *Dr* increased with the increase in τ under different treatments, the interweaving phenomenon of τ points is obvious under different treatments. Overland flow power increased with the increase in rainfall intensity. The relationship between *W* and τ under different litter coverage masses is shown in Table 4. According to the fitting of the *Dr* and *W*, the range of soil anti-erodibility was 0.0085–0.051, and litter coverage enhances soil anti-erodibility. Soil anti-erodibility increased with the increase in litter coverage mass. In the process of litter decomposition (N4–N7–N6–N5), soil anti-erodibility decreased.

**Table 4:** Functional relationships between water flow power (*W*) and soil separation rate (*Dr*).

Treatment	*F*(*x*)	R^2^
N1	*F*(*x*) = 0.0085*x* + 0.0265	0.9333
N2	*F*(*x*) = 0.0219*x* + 0.0243	0.9388
N3	*F*(*x*) = 0.0269*x* + 0.0134	0.9312
N4	*F*(*x*) = 0.051*x* − 0.0025	0.9152
N5	*F*(*x*) = 0.0242*x* + 0.0172	0.8956
N6	*F*(*x*) = 0.0275*x* + 0.014	0.9277
N7	*F*(*x*) = 0.0485*x* − 0.002	0.9864

*W* is water flow power, *Dr is* soil separation rate.

## Discussion

### Influences of litter decomposition on flow pattern of overland flow

**Figure 10:**
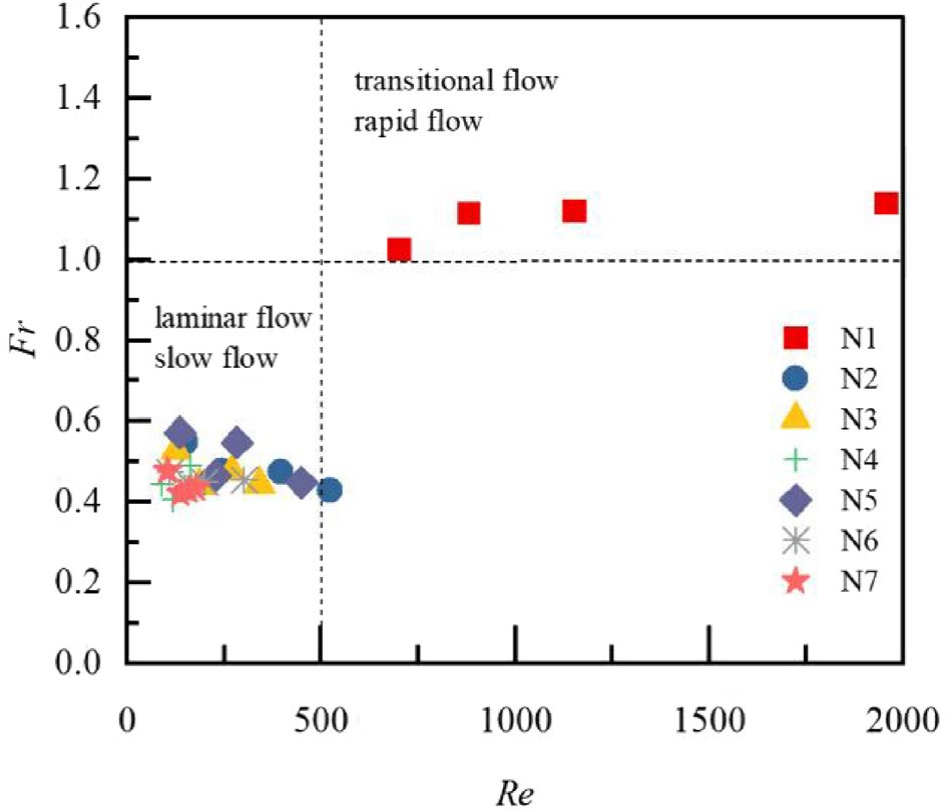
Overland flow regime zoning by litter covering. *Re* is the Reynolds number of the water flow; *Fr* is the Froude constant; T1 is slope 5°, rain intensity 60 mm·h^−1^; T2 is slope 10°, rain intensity 60 mm·h^−1^; T3 is slope 5°, rain intensity 120 mm·h^−1^; T4 is slope 10°, rain intensity 120 mm·h^−1^.

As determined in this study, the water-holding capacity of the semi-decomposed and non-decomposed litter layers was significantly different due to the changes in litter coverage and morphological characteristics^25–29^. The maximum water-holding capacity of the semi-decomposed litter layer is greater compared to the litter non-decomposed layer, which is consistent with previous results^40^. The greater the litter coverage mass, the greater the rainfall interception, which blocked the generation of overland flow and directly affected the runoff discharge. Therefore, discharge is the main factor affecting the characteristics of surface flow force. When litter layer begin to enter the decomposition process, its water-holding capacity will enhance. Thus, the flow pattern changes from transitional flow to laminar flow or from rapid flow to slow flow. In this study, the Re and Fr of overland flow were positively correlated with rainfall intensity and slope (Fig. 10).

The severe rainfall intensity leads to the increase of runoff depth, increases the probability of collision between runoff and underlying surface roughness units, and enhances the overland flow turbulence.According to the flow pattern index *m*, there was no positive correlation between the dynamic turbulence of overland flow and litter coverage mass (Table 3). The litter layer has water retention characteristics, and its substances were easily transported through overland flow. When the litter coverage mass was large, litter was not easily washed away by overland flow and instead accumulated. Given its loose structure, the semi-decomposed litter layer was easy to disperse in the process of rainfall splashing and runoff erosion. The proportion of semi-decomposed litter layer increases with the progress of litter decomposition, underlying surface roughness increase, and the effect of flow regime change is more obvious.

### Influences of litter decomposition on resistance characteristics of overland flow

The increase in litter coverage mass leads to the increase in coverage rate, which increases the contact area for collisions and friction between the overland flow and the litter layer(s), which consumes flow energy and increases the resistance coefficient^41^. The change of *f* with litter coverage mass was not consistent under different slopes and rainfall intensities. When the rainfall intensity was 60 mm·h^−1^, the *f* increased with the increase in litter coverage mass. When the rainfall intensity was 120 mm·h^−1^, the *f* of N3 was greater than in N4, but it was unstable. When the rainfall intensity was large enough, overland flow deposits litter in a short distance^24^. At the same time, with intense rainfall part of the soil on the slope becomes exposed, the runoff depth and resistance coefficients decreased.

In the process of litter decomposition, the *f* decreased. This phenomenon shows that the ability of litter layer to absorb water is decline. When the litter gets into the decomposition state, the depth of runoff is large. While raindrops only affect the overland flow area near the free surface, and the impact on overland flow relative reduces. This study found that the *f* decreased with the increase of *Re*, which is consistent with the findings of previous studies^42^.

### Influences of litter decomposition on soil detachment capacity of overland flow

In the early stage of a rainfall, the topsoil is dry, the infiltration rate of soil is high, and the runoff is small. With an increase in rainfall duration, the runoff increased first, and after that it was stable. This trend was due to the separation of soil particles by runoff under the action of gravitational potential energy and kinetic energy. Therefore, when the runoff was large, the residual energy after overcoming all kinds of resistance was large^29^, and the greater the separation ability of runoff to soil and the greater the *Dr*. In the process of litter decomposition, the *Dr* increased as litter biomass reduction. However, the *Dr* decreases first and then increased in T3 treatment. The effect of the underlying surface on the *Dr* is digest when the rainfall intensity is relatively large.

Comparing the effects of the semi-decomposed litter layer and the non-decomposed litter layer on soil separation rates under the same litter coverage mass, we found that the *Dr* of the semi-decomposed layer was small due to its maximum water-holding capacity being large and overland runoff was therefore small. Under the action of gravity, the semi-decomposed layer of litter is close to the topsoil, which enhances the adhesion of particles on the topsoil and reduces the *Dr*.

In this study, *τ* ranged from 0.004 to 0.013 Pa. The critical runoff shear force τ first increased and then decreased with the increase in litter coverage mass. This trend shows that when the litter coverage go beyond certain range, the litter absorbs rainfall water and produces downward force on the slope, thus reducing the anti-erodibility of soil.

### Using litter morphological characteristics to quantify the litter decomposition effect

In summary, the influence of the litter layer on overland flow is significantly related to runoff flow, rain intensity, slope, and litter coverage mass. The specific relationship is shown in Table 5. When the runoff increases, the flow pattern changes, and the soil separation rate, *Dr*, also increases. The *Re*, *Fr*, *f*, and *Dr* all have a significant power function relationship with litter diameter. Therefore, compared with litter coverage mass, slope, and other factors, the characteristics of litter are the major factors that affect the hydrodynamic parameters of overland flow. When the average diameter of litter leaves is large, the litter layer can effectively reduce the soil separation capacity of overland flow by consuming raindrop kinetic energy and absorbing water. However, the average diameter and biomass of leaves are decreasing in the process of litter decomposition (N4–N7–N6–N5), leading to soil loss.

**Table 5:** Regression relationships between characteristic parameters of slope hydrodynamics and other variables.

*F (x)*	*R*^2^
*Q* = 10^−82.3^*L*^−0.04^*D*^*−*312.8^*S*^18.4^*R*^4.2^	0.902
*Re* = 10^−267.7^*Q*^2.2^*R*^−5.349^*D*^*−*127.9^	0.784
*Fr* = 10^0.57^*M*^−0.001^*D*^*−*0.319^	0.529
*f* = 10^−25.2^*S*^20.9^*M*^0.31^*D*^0.783^	0.780
*Dr* = 10^−10.322^*Q*^0.0001^*L*^−0.0000153^*D*^*−*14.669^	0.946

*Q* is the flow, *Re* is the Reynolds number of the water flow, *Fr* is the Froude constant, *f* is the resistance coefficient, ${D}_{r}$ is the rate of soil separation, *L* is litter length, *D* is litter average diameter, *S* is slope, *R* is rainfall intensity, *M* is litter coverage mass.

## Summary and conclusions

In this study, the effects of litter layer on overland flow hydrodynamic parameters of Masson pine forest were studied. Seven litter layer treatments, two rainfall intensities, and two slopes were explored. The following are the study’s main findings: The semi-decomposed layer’s *Re*, *f*, and *Dr* were lower than that of the non-decomposed layer under the same conditions, which had a significant effect on improving the soil anti-erodibility. In the process of litter decomposition, the *Re* and *Dr* increased, overland flow patterns from transitional flow to laminar flow and from rapid flow to slow flow. Litter coverage, runoff and the diameter of the litter were major parameters that affected the *Re*, *f*, *Fr*, and *Dr*. Shrubs with wide leaves should be selected for understory vegetation replanting. Consequently, this work has enhanced our understanding of runoff process and its controlling factors. Further studies should focus on the effect of litter layers on raindrop splash erosion.

## Data Availability

The data that support the findings of this study are available from the corresponding author upon reasonable request.

## Abbreviations

T1: Slope 5° and rainfall intensity 60 mm·h^−1^
T2: Slope 10° and rainfall intensity 60 mm·h^−1^
T3: Slope 5° and rainfall intensity 120 mm·h^−1^
T4: Slope 10° and rainfall intensity 120 mm·h^−1^
*Re*: Reynolds number
$$Fr$$: Froude constant
$$h$$: Runoff depth
$$f$$: Resistance coefficient
*τ*: Shear force
*W*: Flow power
*D*_*r*_: Rate of soil separation
